# Internal Quality Assurance Program for ERBB2 (HER2) Testing Improves the Selection of Breast Cancer Patients for Treatment with Trastuzumab

**DOI:** 10.1155/2012/261857

**Published:** 2012-02-14

**Authors:** Vittoria Martin, Antonella Camponovo, Morena Ghisletta, Massimo Bongiovanni, Luca Mazzucchelli

**Affiliations:** Institute of Pathology, via in Selva 24, 6600 Locarno, Switzerland

## Abstract

International guidelines for ERBB2 (HER2) testing procedures in breast cancer patients highlight the importance of external quality control. In contrast, internal quality assurance programs have been poorly defined, and their clinical significance has not yet been investigated. We developed a quality assurance scheme by performing HER2 FISH on 724 patients randomly selected out of 1996 patients with breast cancer presenting at our institute. We collected samples monthly for tissue microarray analysis and correlated HER2 gene status with IHC scores. The concordance was excellent (*κ* = 0.92, *P* < 0.0001). HER2 amplification characterized 25% of score 2+ but also 13% of score 1+, thus expanding the number of patients eligible for trastuzumab. Based on these findings, the FISH test is now recommended at our institution for score 1+ and 2+ patients. Adherence to internal assurance program improves patient selection and may lead to the definition of in-house tailored diagnostic algorithms different from those proposed in international guidelines.

## 1. Introduction

ERBB2 (commonly referred to as HER2) protein overexpression or gene amplification is a predictive marker for response to trastuzumab (Herceptin) in patients with breast cancer (BC) [1, 2]. Immunohistochemistry (IHC) and fluorescence in situ hybridization (FISH) are the only tests currently approved by the Federal Drug Administration (FDA) and by the European Medicines Agency (EMA) for HER2 testing. In clinical practice, patients may be considered eligible for treatment with trastuzumab if classified as score 3+ by IHC or if they carry gene amplification as detected by FISH. In contrast, patients showing score 0 or 1+ by IHC or the absence of gene amplification by FISH should not be submitted to targeted therapy. The American Society of Clinical Oncology and the College of American Pathologists (ASCO/CAP) strongly recommend that patients with equivocal results by IHC (i.e., score 2+) should be further investigated by FISH to confirm eligibility [3].

The importance of external quality proficiency assessment for HER2 testing has been emphasized by the ASCO/CAP and by the United Kingdom National External Quality Assurance Scheme (UK-NEQAS) [4–6]. In contrast, although strongly recommended by the ASCO/CAP, internal quality assurance programs have been weakly defined and their clinical significance has been poorly investigated. Thus, the aim of this study was to develop a reliable and effective internal quality assurance scheme and to determine its influence on patients management.

## 2. Materials and Methods

### 2.1. Patients

**Figure 1:**
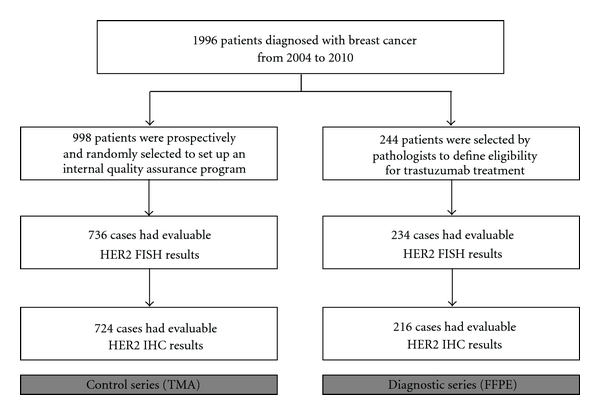
Breast cancer patient selection chart.

From January 2004 to December 2010, a total of 1996 patients were diagnosed with breast cancer at the Institute of Pathology, Locarno, Switzerland. Formalin-fixed paraffin-embedded (FFPE) tissue samples of 998 cases (50%) were prospectively selected through a systematic random sampling procedure (sampling interval = 2) and were used to set up an in-house internal assurance system (Figure 1). Tissue samples were collected monthly and included on tissue microarrays (TMA). Every patient was punched twice on TMA. Only cases for which at least one core was evaluable were considered. 169 (17%) patients were excluded from the study because of inadequate sampling on the TMA, such as missing or insufficient/detached tumor tissue, or presence of necrosis. 93 (9%) patients were excluded due to inadequate FISH results (absence or low efficiency of hybridization, poor signals quality, presence of autofluorescence, and presence of artifacts).

In 736 (74%) cases FISH analysis for HER2 was considered reliable. In 12 cases, data on HER2 protein expression as determined by IHC were not available. Eventually, the “control series” consisted of 724 cases, corresponding to 36.3% of the total number of patients with invasive breast cancer diagnosed over the observation period. The mean age of patients was 63 years, the mean tumor size was 21.6 mm, estrogen receptor expression (defined as more than 5% of positive cells) was detected in 85% of cases, and progesterone receptor expression was detected in 70% of cases. The histotypes were ductal (74.9%), lobular (10.4%), and other (14.7%). The histological grade was well to moderately differentiated in 62% and poorly differentiated in 38% of cases. Patient and tumor characteristics were comparable to data obtained in a recent population-based study from the same geographical area [7], thus indicating that the control series was representative of the general population living in our region.

We also investigated whole tissue samples of 244 breast cancer patients that were selected by pathologists, on the basis of ambiguous or not representative immunohistochemical profile, or by oncologists, on the basis of clinical features and indications for trastuzumab treatment. Similarly to the control series, we considered only patients for which both FISH and IHC results were available (Figure 1). 10 (4%) patients were excluded for unsuccessful FISH experiments and 18 (7%) patients for not evaluable IHC staining. Eventually, 216 cases were evaluated in this group, which was referred to as the “diagnostic series.” Forty-seven cases from this series were also included in the control series.

### 2.2. Construction of TMAs

TMAs were constructed as described previously [8]. Every month, paraffin blocks used for immunohistochemical analyses in the routine diagnoses of breast cancers were selected through a systematic random sampling procedure (sampling interval = 2) by technicians unaware of clinical data and tumor characteristics and were included on a TMA. For each patient, two representative cores 0.6 mm in diameter were punched. Each tumor array contained samples of approximately 10 to 30 patients. As an internal control, two previously characterized breast cancer specimens were included in each TMA, one with and one without HER2 gene amplification.

### 2.3. Fluorescence In Situ Hybridization

FISH was performed on 4 *μ*m thick sections of TMAs of the control series and on whole FFPE tissue sections of the diagnostic series using the FDA-approved LSI HER2/CEP17 probe set (PathVysion, Abbott, Baar, Switzerland). Slides were treated according to the manufacturer's instructions. FISH was evaluated according to the established guidelines and by calculating the ratio of HER2 signals to CEP17 signals (*R*) [3]. Patients were stratified depending on their HER2 gene status as amplified (*R* > 2.2), not amplified (*R* < 1.8), and equivocal (1.8 < *R* < 2.2).

### 2.4. Immunohistochemistry

IHC was performed on 4 *μ*m thick FFPE tissue sections using the FDA-approved clone CB11 (Novocastra, Newcastle upon Tyne, UK) according to the manufacturer's instructions, which were adapted to an internal standardized protocol for automated staining (Benchmark, Roche, Basel, Switzerland). Because HER2 IHC is routinely performed on all BC samples obtained at our institute, overexpression was evaluated on full FFPE tissue sections in both the control series (on the original block selected for TMA punching) and the diagnostic series. As an internal control, a breast cancer specimen with a known HER2 score 3+ was included on each histological slide subjected to immunohistochemical analysis. Patients were classified according to their HER2 protein staining as score 0, 1+, 2+, or 3+, according to well-established evaluation criteria [3].

### 2.5. Concordance

Cohen's kappa test (*κ*) was used to evaluate the concordance between the IHC and FISH results. This rate was calculated considering negative cases, that is, scores 0 and 1+ by IHC and no amplification as determined by FISH, and positive cases, that is, a score 3+ by IHC and amplification as determined by FISH. Patients with equivocal IHC or FISH results (score 2+ or equivocal) were not considered for this purpose. Concordance was assessed by Fleiss' equally arbitrary guidelines, which characterize *κ* values over 0.75 as excellent, 0.40 to 0.75 as fair to good, and below 0.40 as poor [9]. All statistical tests were two-sided. Significance levels were set at *P* ≤ 0.05. All statistical analyses were carried out using the SAS version 9.1 (SAS Institute Inc, Cary, NC).

## 3. Results

In the control series, 128 (18%) out of 724 patients were classified as score 3+ by IHC, 51 (7%) patients as score 2+, 60 (8%) patients as score 1+, and 485 (67%) patients as score 0 (see Table 1). Using FISH analysis, 153 (21%) out of 724 patients were classified as amplified, 13 (2%) patients as equivocal, and 558 (77%) as not amplified (see Table 1). The percentage of concordant results between IHC and FISH was equal to 97.3%, with a statistically significant *κ* value of 0.92 (*P* < 0.0001).

Eighteen (2%) patients showed discordant HER2 protein status as detected by IHC and HER2 gene status as detected by FISH: 7/485 (1%) patients classified by IHC as score 0 and 8/60 (13%) patients classified as score 1+ showed gene amplification by FISH, whereas 3/128 (2%) patients with score 3+ by IHC did not have HER2 gene amplification. To investigate the discrepancies for these 18 patients, all IHC stainings were reevaluated, and FISH was repeated on whole FFPE tissue sections. In 11 (61%) out of these 18 patients, the discrepancy was due to pathologist error in scoring of HER2 protein expression or of HER2 gene status, in 5 (28%) cases, protein or genetic tumor heterogeneity was considered to be the source of the disagreement between IHC and FISH, and, in 2 (11%) patients, no reasons for the discrepancy were found, as the FISH and IHC analyses were truly discordant.

In the diagnostic series, 21 (10%) out of 216 patients were classified as score 3+ by IHC, 134 (62%) patients as score 2+, 39 (18%) patients as score 1+, and 22 (10%) patients as score 0 (see Table 2). Using FISH, 59 (27%) out of 216 patients were classified as amplified, 23 (11%) patients as equivocal, and 134 (62%) as not amplified (see Table 2). The percentage of concordant results between IHC and FISH was equal to 94.8%, with a statistically significant *κ* value of 0.88 (*P* < 0.0001). The 4 (2%) discordant cases were classified as score 1+ by IHC and showed gene amplification by FISH. Disagreement was due to tumor heterogeneity in 3 (75%) patients and to an error in the scoring of the IHC staining in one (25%) patient.

Differences in the frequencies of the IHC and FISH categories between the control series and the diagnostic series were related to the type of BC patients included in the two cohorts (randomly selected patients versus patients selected on demand). However, when grouping patients by HER2 FISH status, IHC scores between the two series overlapped (Tables 1 and 2). As an example, considering FISH amplified category in the control series and in the diagnostic series, respectively, 0% and 1% of cases were classified as score 0, 13% and 10% were classified as score 1+, 25% and 25% were classified as score 2+, and 98% and 100% were classified as score 3+, thus demonstrating the high reproducibility of this scheme at our institute.

## 4. Discussion

Accurate determination of HER2 status is essential for optimal patients selection for target therapy. Testing procedures follow international guidelines, and the importance of external quality assurance programs has been widely recognized. Experts propose two opposite work flows: the use of the IHC test for initial evaluation followed by FISH only on equivocal cases (score 2+) or, otherwise, the use of FISH as the frontline assay [3, 4]. No gold standard exists currently, but it is generally accepted that the detection of gene amplification by FISH is more accurate and predictive for the response to trastuzumab therapy in breast cancer patients [4]. It has been suggested that performing FISH routinely as the initial test is a cost-effective approach, considering the therapeutic context as a whole [4, 10]. In daily practice, however, many pathology laboratories still perform IHC as the frontline test because of technical aspects, cost, and economic issues related to the Health Insurance System.

Internal quality assurance programs for HER2 testing have been poorly defined, and their significance in the clinical management of BC patients remains unknown. The ASCO/CAP guidelines recommend initial validation procedures before the test is offered, ongoing biannual test validation, ongoing equipment maintenance, the use of standardized operating protocols with the routine use of control materials, and ongoing education and training of laboratory personnel and pathologists [3].

To provide consistent and reproducible diagnostic results, we developed an internal quality assurance scheme by performing HER2 FISH on a representative series of BC patients, randomly and prospectively included on TMAs, and by correlating HER2 gene status with IHC scores. This approach is robust, cost-effective, and not time-consuming. It allows continuous monitoring of the concordance between IHC and FISH and ongoing reciprocal controls for both techniques.

Our laboratory follows the recommendation for HER2 testing mentioned above and formulated by the ASCO/CAP [3]. In particular, optimal IHC and FISH testing requirements and optimal tissue handling requirements have been implemented since 2005. Test validation assays have been performed, and both IHC and FISH procedures are performed using appropriate positive and negative controls. The pathologists scoring the immunohistochemical staining and the cytogeneticists reading the FISH results undergo ongoing competence assessment and education. External quality assurance programs were routinely carried out during the study period (UK-NEQAS and others). Our laboratory has been accredited since 2009 by the Swiss Accreditation System (SAS) according to the ISO/IEC 17025 and ISO/IEC 15189 norms (STS 525). Nevertheless, the implementation of an internal quality assurance scheme allowed us to modify the diagnostic algorithm suggested by international guidelines to identify patients eligible for trastuzumab. In fact, in our series, 13% of patients considered negative by IHC testing (i.e., score 1+) had HER2 gene amplification. We therefore advise clinicians referring to our laboratory to require FISH as a predictive test not only in BC patients with IHC score 2+ but also in score 1+ patients. In contrast, we consider FISH analysis of score 0 and score 3+ samples not to be useful.

There are at least two main reasons to explain the “underperformance” of IHC in detecting patients with HER2 amplification in score 1+ cases. First, the standardization of IHC tests remains sometimes problematic and may be affected by preanalytical and analytical factors [3]. Even adherence to external quality assurance programs for IHC HER2 testing may be not enough to obtain reproducible results because, in contrast to ongoing internal quality assurance scheme, such external programs pursue quality control only sporadically. Furthermore, the results may be biased by handling samples with awareness by laboratory personnel and pathologists, thus not truly reflecting daily work procedures. Second, our results underscore the well-known poor reproducibility of the evaluation of semiquantitative immunohistochemical staining in equivocal cases [3, 4]. In contrast, we do not think that the implementation of validated IHC tests would have led to more precise HER2 testing because the concordance between the “in-house” results and FISH analysis was excellent. Furthermore, external quality controls of HER2 testing by IHC always yield positive feedback.

In conclusion, we strongly believe that an ongoing internal quality assurance program for HER2 testing improves performance, reduces the frequency of inaccurate results, and may have more clinical impact than sporadic external quality controls. We suggest that pathology laboratories should monitor HER2 testing continuously by correlating IHC and FISH and that analysis of these data may lead to the definition of in-house tailored diagnostic algorithms different from those proposed and recommended in the literature.

## Figures and Tables

**Table 1:** Results of HER2 protein status as determined by IHC and gene status as determined by FISH in breast cancer patients from the control series.

		FISH							
Control series		Not amplified		Equivocal		Amplified		Total	
IHC	0	471	97%	7	1%	7	1%	485	67%
	1+	51	85%	1	2%	8	13%	60	8%
	2+	33	65%	5	10%	13	25%	51	7%
	3+	3	2%	0	0%	125	98%	128	18%

	total	558	77%	13	2%	153	21%	**724**	100%

**Table 2:** Results of HER2 protein status as determined by IHC and gene status as determined by FISH in breast cancer patients from the diagnostic series.

		FISH							
Diagnostic series		Not amplified		Equivocal		Amplified		Total	
IHC	0	22	100%	0	0%	0	0%	22	10%
	1+	30	77%	5	13%	4	10%	39	18%
	2+	82	61%	18	13%	34	25%	134	62%
	3+	0	0%	0	0%	21	100%	21	10%

	total	134	62%	23	11%	59	27%	**216**	100%
